# Towards a new era in medicine: therapeutic genome editing

**DOI:** 10.1186/s13059-015-0859-y

**Published:** 2015-12-22

**Authors:** Matthew H. Porteus

**Affiliations:** Department of Pediatrics, Stanford University, Welch Road, Stanford, CA 94305 USA

## Abstract

Genome editing is the process of precisely modifying the nucleotide sequence of the genome. It has provided a powerful approach to research questions but, with the development of a new set of tools, it is now possible to achieve frequencies of genome editing that are high enough to be useful therapeutically. Genome editing is being developed to treat not only monogenic diseases but also infectious diseases and diseases that have both a genetic and an environmental component.

## The potential therapeutic importance of genome editing

In 1901, Sir Archibald Garrod identified alkaptonuria as the first known human genetic disease. Today, we recognize that there are at least 8000 human diseases that are caused by mutations in single genes (monogenic diseases); the number increases almost every day [1, 2]. While all of these diseases are classified as ‘rare’ in the US because they affect fewer than 200,000 people, they may affect over 400 million people worldwide. Some, such as sickle cell disease, affect tens of millions of people around the world and are only ‘rare’ in certain parts of the world, including the US, Europe and far east Asia. For a tiny subset of patients, allogeneic hematopoietic stem cell transplantation (allo-HSCT) or solid organ transplantation can be used to cure their genetic disease, but for the vast majority of patients there is no cure and at best they are treated by management of symptoms.

Therapeutic genome editing was born out of the idea that the ideal therapy for monogenic diseases would be to develop a method that can correct the disease-causing mutations directly; but as genome editing has developed in concert with continuing improvements in our understanding of the genetic contribution to non-monogenic diseases, the principle of genome editing is being developed not only to cure monogenic diseases but also to cure more common diseases that have multifactorial origins. The use of genome editing to cure monogenic disease is conceptually simple (genome editing can be used to correct the underlying genomic typographical errors), but the power of genome editing is that it provides a mechanism that can do more than simply modify single nucleotides. It is a method that can make more sophisticated and nuanced genomic changes, which can be used to cure more common diseases or to modify their course.

The exact nature of the therapeutic edit has to be driven by a solid understanding of the interplay between the underlying genetics and the specific pathophysiology of the disease. That is, one editing strategy might be appropriate for one disease but not applicable to another. This review will describe the basic strategies of genome editing and the tools that are now available both to correct typographical errors and to make more sophisticated changes to the genome. I will then discuss how genome editing is being developed to treat genetic, infectious, and acquired diseases. Finally, I end with a brief discussion of issues surrounding the use of genome editing in situations that might cause the engineered genetic change to be passed from one generation to the next.

## The development of genome editing and the contemporary toolbox

Genome editing, also previously known as gene targeting, has been a powerful research tool for scientists. In particular, the ease of gene targeting in yeast was one factor that made yeast such an important model organism in studies of the pathophysiology of human disease [3, 4]. The importance of gene targeting as a research tool was further highlighted by the awarding of the Nobel Prize in Physiology or Medicine in 2007 to Drs Oliver Smithies and Mario Capecchi for their development of gene targeting in mouse embryonic stem cells and for their subsequent precise genetic engineering of mice—a transformational advance in understanding human pathophysiology [5, 6]. Even in the earliest days of gene therapy, it was recognized that genome editing might be the ideal approach for curing genetic diseases, but the earliest studies were stymied by the low absolute frequency of gene correction by homologous recombination in human somatic cells (10^−6^) [7–9]. A critical breakthrough was the discovery that by creating a site-specific DNA double-stranded break (DSB) in the target gene it is possible to stimulate genome editing by homologous recombination by 2–5 orders of magnitude, providing overall frequencies of 5 % or more [10–13]. In addition to stimulating gene targeting by homologous recombination by five orders of magnitude, a site-specific DSB could stimulate mutations such as small insertions/deletions at the site of the DSB by nine orders of magnitude. Thus, the DSB became a key principle in the development of genome editing.

**Fig. 1:**
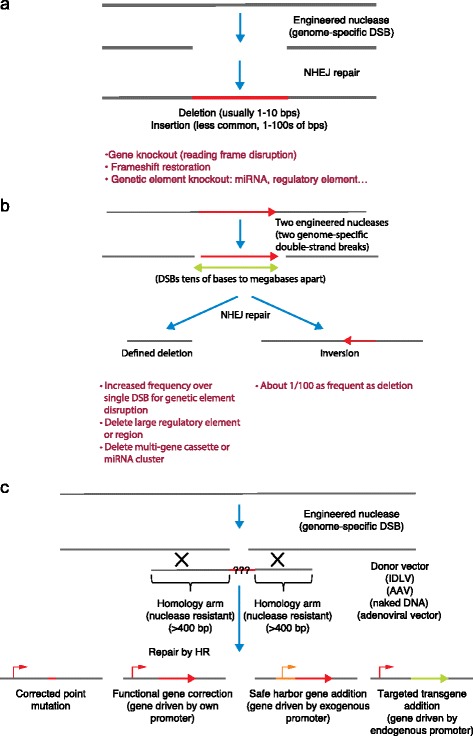
Nuclease-based genome editing creates a specific double-stranded break (*DSB*) in the genome and then allows the cell’s own endogenous repair machinery to repair the break. **a** When the editing of a single break occurs by nonhomologous end-joining (*NHEJ*), insertions/deletions are created at the site of the break. **b** When the editing of two DSBs occurs by NHEJ, chromosomal deletions, inversions or translocations can be created. **c** When editing of a single DSB occurs by homologous recombination (*HR*) using a provided donor sequence, precise changes in the nucleotide sequence ranging from a single base insertion to the introduction of a large cassette of genes can occur. Using NHEJ and HR mediated editing, it is now possible to inactivate genetic elements, create defined deletions ranging from a few bases to thousands of bases, and precise nucleotide changes to the sequence of the genome. *AAV* Adeno-associated virus, *bps* Basepairs, *IDLV* Integrase-deficient lentivirus

The basic process of nuclease-based genome editing is to create a specific DSB in the genome and then allow the cell’s own endogenous repair machinery to repair the break (Fig. 1). The cell can repair the break using one of two basic mechanisms: nonhomologous end-joining (NHEJ) or homologous recombination (HR) (see Box 1; Fig. 1) [14–17]. When the editing of a single break occurs by NHEJ, insertions/deletions are created at the site of the break [17] (Fig. 1a). The size of deletions tends to be larger than that of insertions, except when extrachromosomal DNA is captured at the site of the break (a rare but measurable occurrence), in which case insertions of hundreds of basepairs (bp) can occur [18, 19]. When editing of a single break occurs by HR using a provided donor sequence, precise nucleotide changes in the genome range from a single base insertion to the introduction of large cassette of genes (Fig. 1c) [20, 21]. When the editing of two breaks occurs by NHEJ, chromosomal deletions, inversions or translocations can be created (Fig. 1b) [22]. These gross chromosomal rearrangements can be generated intentionally for therapeutic purposes, but they also must be evaluated because any nuclease platform has the potential to produce off-target effects.

In seminal studies of the importance of DSBs, researchers used an artificial system in which the target site for a natural homing endonuclease (also sometimes called a ‘meganuclease’), I-SceI, was engineered into the genome of somatic cells; the frequency of genome editing was measured at that engineered I-SceI site [9, 13, 23–25]. The barrier to high-frequency editing was that neither I-SceI nor the other meganucleases could be easily re-engineered to recognize natural target sites in the genome. The first solution to this problem was the development of zinc-finger nucleases (ZFNs) (originally called ‘chimeric restriction enzymes’, then called ‘chimeric nucleases’) [26–28]. ZFNs are artificial proteins in which a zinc-finger DNA-binding domain is fused to the nonspecific nuclease domain derived from the FokI Type II S restriction endonuclease. At first, engineered ZFNs were shown to be as efficient as I-SceI in stimulating gene targeting in human somatic cells. Then, because the zinc-finger DNA binding domain can be engineered to recognize new target sites, ZFN-based protocols became the first method used to stimulate genome editing in human somatic cells to frequencies that are therapeutically relevant [9, 29, 30]. This work in human somatic cells paralleled the important work by Dana Carroll and his colleagues, who showed that ZFNs could be used to edit the complex genome of eukaryotic *Drosophila melanogaster*, both by mutagenic NHEJ and by HR [17, 31]. For a number of years, the only engineered nucleases in the genome editing toolbox were ZFNs [32, 33] and re-engineered meganucleases [34]. In the past 5 years, however, the development of TAL effector nucleases (TALENs) [35–37], CRISPR/Cas9 nucleases [38–40], and hybrid nuclease platforms [41–43] have dramatically expanded the engineered nuclease toolbox.

There are four basic and two hybrid engineered nuclease platforms that include engineered meganucleases, ZFNs, TALENs, CRISPR/Cas9 nucleases, mega-TAL nucleases, and Cas9-FokI nucleases (Box 2; Table 1). There are subtle differences between each of these nuclease platforms—for example, the type of break that is created is different: meganucleases and mega-TALs generate breaks with 3′ overhangs; ZFNs create breaks with 5′ overhangs; TALENs create breaks that are variable in position that are usually (but not always) 5′ overhangs, as determined by the properties of the FokI nuclease (Fn); and CRISPR/Cas9 nucleases create blunt breaks. In general, however, each of these platforms mediates their editing effects through the creation of a DSB and thus they share a fundamental mechanism of action.

**Table 1:** Contrasting characteristics of the four standard nuclease platforms

Nuclease	Target site length	Mechanism of recognition	First use in human cells	Ease of design	Number of components	Size of mRNA transcript
Engineered meganuclease	>18 bp	Protein-DNA	1994 (I-SceI)	Extremely difficult	1	Short
Zinc-finger nuclease	18–36 bp	Protein-DNA	2003	Difficult	2	Short
TAL effector nuclease	24–40 bp	Protein-DNA	2011	Easy	2	Long
CRISPR/Cas9 nuclease	19–22 bp (*Streptococcus pyogenes* Cas9)	RNA-DNA Watson-Crick base-pairing	2013	Simple	1 (if using a complex guide RNA with Cas9 protein) or	Long
					2 (if delivering guide RNA and Cas9 separately)	

The only tool needed for NHEJ-mediated genome editing is an engineered nuclease, but HR-mediated genome editing also requires an engineered donor vector. Donor vectors can be designed to template single bp changes or to insert large multi-gene cassettes into the genome. The homology arms for nuclease-mediated genome editing can be much shorter than those required for HR-mediated gene targeting in murine embryonic stem cells: instead of having to be 10 kilobases or greater, they can be as short as 400 bp [18]. Shortening the homology arms to below 400 bp does, however, seem to decrease the overall editing efficiency. Single-stranded oligonucleotides (ssODNs) have also been used to template small nucleotide changes after the induction of a DSB [44]. The ease with which ssODNs can be synthesized makes this approach relatively accessible to the researcher, but the mechanism by which ssODNs create a targeted change in the genome does not rely on the classic HR pathway and is not well understood. Moreover, ssODNs induce a replication and cell cycle arrest even in cancer cell lines [45] and would probably be even more problematic in primary therapeutically relevant human cell types, as demonstrated in part by the work of Hoban et al. [46].

## Issues of delivery and process development

A mantra in the field has been that the three most important issues in gene therapy are delivery, delivery, and delivery. As the toolbox for genome editing has expanded, that mantra now also applies in many respects to therapeutic genome editing: what is the optimal process to deliver highly active genome-editing reagents into the most clinically relevant cell type? The answer to this question is increasingly disease specific. An important consideration in determining an appropriate delivery strategy is that genome editing, in contrast to gene-augmentation strategies, is a hit and run approach. In fact, sustained expression of the nuclease not only is not needed but should be avoided: continued expression of a nuclease increases the probability of deleterious genomic instability and may either compromise the edited cell’s fitness or predispose the exposed cell to transformation.

For ex vivo manipulation of cells, standard nonviral delivery of the nuclease as RNA, or ribonucleoprotein (RNP) for the CRISPR/Cas9 system, seems to be the most promising approach. Delivering the nuclease component as RNA or RNP ensures that both the activation of the Type I interferon response and the duration of the expression are minimized. The RNA or RNP can be delivered into a cell by a variety of mechanisms that are determined by the specific cell type’s ability to be transfected with different complexes. A universal method of delivery that is effective across all cell types is electroporation, in which cells are mixed with the RNA or RNP and a brief electrical pulse is passed through the mixture, thereby creating membrane holes through which the RNP or RNA enters. Multiple different electroporation devices are now available and, amazingly, electroporation conditions can be found that create minimal cellular toxicity as long as DNA or other nucleic acids that activate the innate immune system are not included in the mixture. For applications that simply require the delivery of the nuclease, this seems to be a robust solution. For applications that require HR-mediated editing, a DNA molecule also needs to be delivered. Delivery of naked DNA into cancer cell lines has been an effective method to deliver the donor vector, but the delivery of naked DNA into primary cells, particularly T cells and hematopoietic stem and progenitor cells, activates a deleterious innate immune response that both decreases the frequency of genome editing and compromises the fitness of the edited cell. Using adeno-associated virus (AAV) to deliver the donor template into cells may be a solution to this problem because AAV, like many viruses, has evolved to escape recognition by the innate intracellular immune response [47–50].

For therapeutic applications that require in vivo editing of cells, the challenge is greater and a solution has not been determined. Again, the solution to the in vivo delivery problem may differ depending on which target cell type needs to be identified. The solution for editing hepatocytes, for example, will probably be different than that for editing muscle, which will be different again from that for editing cells in the central nervous system. Nonetheless, with the development of multiple different serotypes of AAV that preferentially transduce different cell types in vivo [51, 52], the development of new methods of delivering mRNAs to cells, and the increasing sophistication of nanoparticles (both lipid and nonlipid based) to deliver to specific tissues, there are likely to be solutions forthcoming soon. Developing a delivery method in which the nuclease is not expressed for a sustained period of time is important both from a genotoxic standpoint and from an immunologic standpoint. It should be anticipated, until proven otherwise, that all of the engineered nuclease platforms will be seen by the immune system as foreign and will elicit a robust immune response that will both eliminate the therapeutically edited cells and perhaps cause toxic organ damage.

As therapeutic genome editing gathers momentum, an increasing number of innovative approaches are being developed. These can be classified along three different axes: NHEJ- vs. HR-mediated genome editing; ex vivo vs. in vivo delivery; and suitability for genetic vs. infectious vs. nongenetic diseases. Examples of some of these various strategies are discussed below.

## Potential therapeutic applications of nonhomologous end-joining mediated genome editing

Diseases that can be addressed using NHEJ-mediated genome editing are those in which mutating a genetic element, whether a coding region, a regulatory element, or some other genetic element, might result in clinical benefit. One example of this approach is to delete the erythroid enhancer for *Bcl11A* in hematopoietic stem/progenitor cells (HSPCs) in order to upregulate γ-globin to treat sickle cell disease and β-thalassemia [53–55]. Both sickle cell disease and β-thalassemia are monogenic diseases caused by mutations in the *HBB* gene. Both diseases could be cured if *HBG,* a gene closely related to *HBB*, could be upregulated such that it could either replace the missing γ-globin protein (in β-thalassemia) or counteract the dysfunctional γ-globin protein (in sickle cell disease). Studies of the globin switch have demonstrated that *Bcl11-A* is a transcriptional repressor of *HBG* and that repression of *Bcl11A* results in the de-repression of *HBG* [56]. Moreover, when used as a research tool, genome editing demonstrated that deletion of a specific regulatory element in the *Bcl11A* gene, the erythroid enhancer, could repress *Bcl11A* in the erythroid lineage but not in the B-cell lineage, thus validating the inactivation of this element by NHEJ-mediated genome editing in HSPCs as a therapeutic strategy [53].

A different strategy using NHEJ-mediated genome editing is being developed to treat Duchenne’s muscular dystrophy, a monogenic disease cause by mutations in the *Dystrophin* gene. In this in vivo strategy, a single nuclease might be delivered into muscle fibers to create an insertion/deletion that compensates for the original frameshift mutation (thus reverting the pathologic reading frame mutation). Alternatively, a pair of nucleases might be delivered into muscle fibers to delete a set of exons in order to delete pathologic mutations, thereby converting Duchenne’s muscular dystrophy to the less severe Becker’s muscular dystrophy. Proof-of-concept studies have been published for both of these strategies, but the challenge that remains is to achieve the desired editing in a fraction of muscle fibers, including the heart and diaphragm tissues, large enough to alter the clinical course of the disease significantly [57–59]. In addition, as a general principle, any disease that might be treated by RNA interference (RNAi)-mediated knockdown of a gene [60, 61] might be more definitively treated by genome editing. Editing would provide permanent knockdown of the gene and therefore would not require repeated dosing of knockdown RNAi reagent.

For infectious diseases, ex vivo NHEJ-mediated genome editing has already reached Phase II clinical trials as a method to generate a T-cell population that is resistant to HIV infection. These studies are based on the discovery that people with bi-allelic mutations in the *CCR5* gene are near completely resistant to HIV infection, and on the cure of an HIV patient by allo-HSCT using a donor whose stem cells contained a bi-allelic mutation in the *CCR5* gene [62]. Sangamo Biosciences and their collaborators have engineered ZFNs to target the *CCR5* gene, and then used these ZFNs to mutate the *CCR5* gene in primary T cells derived from patients already infected with HIV [63–65]. In Phase I trials, they demonstrated that this approach was both feasible and safe, and Phase II trials are now underway [65].

In vivo NHEJ-based genome-editing approaches are also being developed for infectious diseases. In multiple proof-of-concept studies, nucleases have been engineered to recognize key elements of viral genomes (including those of HIV and hepatitis B) in order to create mutations that will inactivate the virus [66–68]. These studies have demonstrated that such nucleases can be engineered and that they can alter viral kinetics in in-vitro models, but real challenges remain in how to apply this strategy in an in vivo setting where delivery to nearly all infected cells must be achieved and in a way that does not require constitutive expression of the nuclease.

Finally, NHEJ-mediated genome editing has been applied in a proof-of-concept study as a potential approach to treat high cholesterol. *PCSK9* is a regulator of cholesterol and those who have the rare homozygous deficiency in *PCSK9* are otherwise healthy but have extremely low cholesterol levels. In vivo nuclease-mediated genome editing has been used to mutate the *PCSK9* gene in livers, with a resultant drop in cholesterol levels [69, 70]. Although there are multiple caveats to these experiments, they do show, in principle, how in vivo editing might be used to treat multifactorial diseases whose course could be modified by using genome editing to create a clinically useful genotype.

## Potential therapeutic applications of homologous recombination mediated genome editing

Deep understanding of the pathophysiology of certain diseases can show how NHEJ-mediated genome editing could be used as therapy for those diseases. In general, however, the ability to harness HR-mediated genome editing both ex vivo and in vivo has the potential to impact on an even larger number of diseases.

There are numerous genetic diseases of HSPCs, such as sickle cell disease, β-thalassemia, severe combined immunodeficiency and chronic granulomatous disease, that can be cured by allo-HSCT. In allo-HSCT for these types, the hematopoietic system is replaced by cells containing at least one wild-type version of the gene and, for this reason, some have called it ‘allogeneic gene therapy’ [71]. Using HR-mediated genome editing, it would be possible to replace genetically correct allogeneic stem cells with genetically corrected autologous cells. This can be done either by directly correcting the defective gene [46] or by using HR-mediated genome editing to target the therapeutic transgene to a ‘safe harbor’ [72]—a genomic site in which the transgene would be expressed at the needed levels without causing dysfunction or transformation of the modified cell [73]. One potential issue with gene correction by HR is that many genetic diseases, sickle cell disease being an exception, are caused by mutations throughout the gene. The engineered nuclease toolbox is now such that one might consider designing nucleases for each individual mutation. An alternative approach, however, is to design the donor vector such that, after HR, the integrated transgene would functionally correct all (or most) of the disease-causing mutations [74, 75]. Using this strategy, a single set of reagents could be developed to treat all individuals with the genetic disease—a strategy that would significantly simplify the development and regulatory process.

Proof-of-concept studies for in vivo HR-mediated genome editing have been described in which either the underlying mutant gene was directly corrected or a transgene was integrated into a specific location such that it would be expressed at sufficient levels to rescue the underlying defect [76]. In the direct gene correction strategy, nucleases and donor vectors were delivered to fumarylacetoacetate hydrolase (FAH)-deficient mice. Normally, FAH deficiency causes hepatocyte death but, after delivery of the genome-editing machinery, a small number of hepatocytes were corrected. These corrected hepatocytes then re-populated the remaining liver and rescued the mouse from liver failure. In these experiments, the corrected cells had a tremendous selective advantage over uncorrected cells, and the principle of selective advantage is one that is regularly used by the gene therapy community. In the transgene-targeting strategy, nucleases were used to stimulate the targeted introduction of a therapeutic transgene (either Factor IX or lysosomal storage enzymes) into a locus that drove high levels of expression from hepatocytes [77–79]. In this way, a small number of modified hepatocytes were able to rescue an underlying genetic defect at a systemic level.

Ex vivo HR-mediated genome editing is also being developed as a method to create an HIV-resistant immune system [20]. One of the hallmarks of HIV is its ability to mutate and escape any inhibition, and thus it is possible that simply mutating the CCR5 co-receptor will not be sufficient to confer cellular resistance to HIV. Moreover, many HIV patients have already developed HIV variants that enter cells through the CXCR4 coreceptor and thus would escape any approach that only targets *CCR5*; but using HR-mediated editing, one can simultaneously inactivate *CCR5* while inserting a cassette of antiHIV genes, thereby creating multiple genetic blocks to the HIV lifecycle and inhibiting variants that enter through the CXCR4 co-receptor.

Finally, ex vivo HR-mediated genome editing has been shown in proof-of-concept experiments to be therapeutic for an acquired disease. In these experiments, fibroblasts were engineered by HR to secrete a wound-healing growth factor [80]. When these engineered fibroblasts were implanted into mouse wounds, they hastened wound healing by stimulating vascularization. In principle, this demonstrates that cells can be engineered to secrete therapeutic proteins that rescue nongenetic diseases. This might be applied to wound healing in humans but one can also speculate, for example, that a similar approach might be used to engineer cells, either ex vivo or in vivo*,* to secrete neuroprotective factors to slow or halt neurodegeneration or to facilitate neurogenesis or nerve regeneration after trauma.

## Safety and toxicology

One of the tremendous potential advantages of genome editing, when compared to other methods of permanently altering the genome of cells, is the specificity of the process. Nevertheless, the induction of a DSB through a site-specific engineered nuclease is a critical aspect of gene editing and it is well known that DSBs can generate genomic instability, including chromosomal translocations, chromosome loss and aneuploidy [81]. Thus, a key aspect in the clinical development of nuclease-mediated genome editing is to establish a series of assays that assess the potential safety of the process. Unfortunately, the field is too young for there to be any single assay or set of assays that has been validated as establishing whether an editing process will be safe in humans [82]. Instead, safety and toxicology analysis is assessed according to the following principles: 1) minimizing or eliminating off-target DSBs and the consequent insertions/deletions that may be generated; 2) assessing the functional behavior of edited cells using the best available models; and 3) putting the process of genome editing in the context of the natural genomic instability that occurs continuously in everybody. These fundamental criteria apply to whichever nuclease platform is utilized because each platform works through the creation of a DSB.

There are biased and unbiased approaches to assessing the specificity of a nuclease [83, 84]. Bioinformatic tools that are based on searching for sites that have sequences similar to the intended target site can help to predict which off-target sites should be examined. Once a set of sites is identified, deep sequencing can be used to interrogate those sites to determine whether there are nuclease-generated insertions/deletions at those sites. Given the current error rate of deep sequencing methodologies, the limit of detection for a given site is ~0.01 % (or in 1 in 10,000). Moreover, the bioinformatics algorithms are still in their early development and still do not reliably identify all potential off-target sites. To complement the biased approach using bioinformatics, there are newly developed unbiased tools, including break Guide-seq [85], HTGTS [86], BLESS [70], and Digenome-seq [87]. Other assays use extrachromosomal DNA, including AAV [88], naked DNA plasmids [19], and integration-defective lentiviral vectors [89], to capture breaks and to help assess the specificity of a nuclease. These tools have particular power because they can also identify gross chromosomal rearrangements (like translocations) that are not identified using biased approaches. As chromosomal translocations will be a rare but inevitable consequence of induced DSBs, it would be prudent to avoid targeting genes that are involved in cancer-associated chromosomal translocations at this stage in the development of therapeutic genome editing.

The challenge with these unbiased approaches is that they have been developed in specialized cancer cell lines, which do not have intact DNA-repair pathways, and they need to be adapted to primary clinically relevant cell types with intact DNA repair pathways. Nonetheless, these tools still provide useful information in optimizing the specificity of a nuclease. For example, if these assays reveal that the nuclease has multiple off-target sites, then they suggest that the nuclease should be redesigned to be more specific. For ZFNs, this might entail using the obligate heterodimer structure for the nuclease domain or exploring specific alterations in the amino acids that mediate recognition of the target sequence [90]. For TALENs, this might entail using the obligate heterodimer structure for the nuclease domain or using alternative repeat variable di-residues (RVDs) for the TAL effector recognition domain [91]. For CRISPR/Cas9 nucleases, improved design might entail testing a different guide sequence, or using a truncated guide sequence [92] or a paired-nickase approach [93, 94]. For all of the platforms, specificity is increased by limiting the duration of expression of the nuclease, the pharmacologic equivalent of decreasing the AUC (‘area under the curve’) of nuclease exposure [39, 95, 96].

An approach that complements attempts to identify the potential off-target sites of the nuclease directly is to evaluate the genome-editing process by a more classical functional pharmacology–toxicology approach. This strategy evaluates whether the genome-editing process creates cells that are unable to perform their normal function (for example, the ability of hematopoietic stem cells to reconstitute multilineage hematopoiesis), that transform into cancer cells, or that cause the population to become clonally skewed (a possible harbinger of the creation of cells that might transform over a time frame beyond that which can be measured using current assays) [82].

The biased, unbiased, and functional approaches need to be performed in the therapeutic cell type of interest using the clinical-grade genome-editing process planned because assessment in other cell types, particularly already-transformed cancer cell lines, may not be relevant.

An important principle to keep in mind is that dividing cells are undergoing genomic challenge continuously. It is estimated that every time a cell divides, it must repair 20–40 DNA DSBs, not to mention millions of other types of DNA lesions [97, 98]. The consequence of this natural genomic challenge is that a normal dividing stem cell acquires ~3–30 mutations for each cell division; it is estimated 1 million mutations occur every second in an individual. It has been suggested that gene correction by genome editing of patient-derived induced pluripotent cells, followed by whole-genome sequencing to determine if deleterious mutations have occurred, might be a safer approach. The mutational burden caused by ex vivo expansion from a single cell to a therapeutically relevant cell number (for hematopoietic diseases this is in the order of 50–800 million cells depending on the size of the patient) may, in reality, be more oncogenic than just modifying a large number of somatic cells without being able to sequence the genome of any one cell.

## Challenges of germline genome editing

The development of a powerful genome-editing toolbox, combined with the use of this toolbox to create a wide variety of genetically modified species by zygote injection [16], has raised the possibility that someone might use genome editing in human zygotes to create human beings [99, 100]. This possibility was further highlighted by researchers in China who used the approach in tripronuclear human zygotes (human zygotes that are genetically incapable of developing into a human but are nearly identical in concept to diploid zygotes) [101]. The tripronuclear zygote injection experiments highlighted the inefficiency and unpredictability of the process—findings that would have been predicted by animal experiments using healthy diploid zygotes. These results clearly demonstrate that genome-editing technology applied as zygote injection, even if judged ethically permissible or desirable, is not ready for human application. Nonetheless, these specific experiments and the general concept have generated a large number of headlines in both high-profile journals and the lay press. Which direction the conversation turns remains to be determined, but there are several principles that I hope remain at the forefront. First, the ethical issue should not impede the use of powerful genome-editing tools in research to provide a better understanding of germ cells, germ cell development, and early embryonic development. Second, the discussion should be led by thought leaders from a variety of different fields and should include voices from a broad range of stakeholders, including those families who lives have been impacted over multiple generations by the transmission of devastating genetic diseases through their family tree. Third, there is no single ethical viewpoint that has predominance and an ongoing, iterative process in which new understandings and viewpoints can be incorporated is the desired outcome rather than a defined resolution at a single point in time. Finally, the issue of using genome editing that might result in the transmission of specific genotypes to future generations needs to be put into the context of activities already taking place that similarly affect the genotypic makeup of future generations. Two such examples are pre-implantation genetic diagnosis with selective zygote implantation and curing or helping patients with genetic diseases (an unequivocally good thing) such that they might not pass down their disease-causing mutation to their children.

## Future perspectives

The precision of genome editing and the ability to correct disease-causing typographical errors in the DNA sequence have always made the field conceptually appealing. All of the strategies of genome editing could be achieved using engineered meganucleases and ZFNs, but the challenge of making highly active and specific versions of these tools has limited the number of investigators who were committed to the concept. With the development of TALENs and then CRISPR/Cas9 nucleases, the barrier to entry for investigators was decreased so dramatically that essentially any researcher who is interested can begin to explore their own innovative ideas. With this explosion in interest, the pace of progress has increased exponentially. In the decade since the first use of ZFNs in human cells there has been just one clinical trial, so it is exciting to predict that in the next decade there will be tens (if not more) of genome editing-based clinical trials that will be developed by academics, biotechnology start-ups and pharmaceutical companies.

Nonetheless, there remain important issues to be resolved. These include developing a regulatory framework that is tailored to the underlying technology rather than one that is based on a different therapeutic foundation (such as small molecules or antibody biologics). There is also a need to develop safe and effective mechanisms to deliver the genome-editing machinery to a wide variety of tissues in vivo, including the liver, eye, muscle, heart, and brain. Finally, a flexible and adaptive regulatory framework needs to be developed to take into account the ethical and scientific issues around the potential use of genome editing that might alter the genetics of future generations (‘altering heredity’). This framework needs to take into account the diverse group of stakeholders who are affected by the issue and must respect culturally different perspectives.

## Box 1. The mechanism of DNA double-strand break repair

The cell has two primary double-strand break (DSB) repair mechanisms: nonhomologous end-joining (NHEJ) and homologous recombination (HR) [15, 102, 103] (Fig. 1). In NHEJ, the two ends of the broken DNA are ligated back together (by a ‘stitching’ mechanism). For DSBs that are generated by an engineered nuclease, the NHEJ process has high fidelity with >70 % of the breaks being joined in a precise and nonmutagenic fashion [104, 105]. If the nuclease is still active after rejoining, the nuclease will recut the site, creating another DSB and eventually leading to an insertion/deletion at the site of the break. The size of the insertion/deletion is usually 1–15 basepairs but can be much larger; insertions often incorporate random pieces of DNA that are present in the nucleus [18, 19]. Thus, genome editing by NHEJ whereby mutations are created at specific sites in the genome is an iterative process of break and repair until the target site can no longer be cut by the engineered nuclease.

If two DSBs are created simultaneously on the same chromosome, then the NHEJ machinery will create a deletion between the two sites [22]. The use of two DSBs can increase the frequency of inactivating a genetic element [104] or can be used to delete large genomic regions for therapeutic purposes [57]. When the frequency of a deletion is about 1 %, two simultaneous DSBs will result in the inversion of the sequence intervening between the two DSBs. If two DSBs are simultaneously created on different chromosomes, then chromosomal translocations can be created [106, 107]. Such induced chromosomal translocations are useful as a research tool and must be considered when evaluating the safety of a therapeutic genome-editing strategy.

In HR, the cell identifies a piece of DNA that has homology to the site of the DSB and then uses that homologous undamaged DNA as a template in a ‘copy and paste’ mechanism. The template DNA for HR is usually the undamaged sister chromatid. Rarely, the template is the undamaged chromosomal homolog (leading to loss of heterozygosity). In genome editing, the template is an introduced piece of DNA, called a ‘donor’. The donor can be engineered such that when it is used as a template by the HR machinery, single nucleotide changes or multikilobase nucleotide changes are introduced into the genome. In summary, genome editing by NHEJ results in a mutation that has a spatially precise genomic location, whereas genome editing by HR results in a genomic change whose location and nucleotide sequence can both be specified.

The frequencies of NHEJ- and HR-mediated genome editing vary from experimental system to experimental system. In general, NHEJ-mediated editing is more frequent than HR-mediated editing, but when HR-mediated editing is optimized, its frequency can exceed that of NHEJ-mediated editing, even without the use of small molecules [18]. In addition, the relative frequencies of NHEJ- and HR-mediated editing can be altered by small molecules or by RNAi which in some cases work by inhibiting the canonical NHEJ pathway but in other cases through a mechanism that is not well understood, but that these perturbations might result in increased off-target genomic instability and may not be therapeutically useful [108].

## Box 2. Engineered nuclease platforms

**Engineered meganucleases** are derived from the large family of natural homing endonucleases (hundreds of members) [109]. A small number of these endonucleases have been designed to recognize natural target sites in the genome using a variety of strategies, including structure-based design and yeast surface display [34, 110]. Natural meganucleases have historically been the gold standard for specificity, but the challenge of engineering meganucleases for novel target sites has limited their translational development. Furthermore, the specificity of engineered meganucleases has not been fully evaluated.

**Zinc-finger nucleases (ZFNs)** are artificial proteins in which a zinc-finger DNA-binding domain is fused to the nonspecific nuclease domain from FokI [28, 33]. As the nuclease domain needs to dimerize in order to cut DNA efficiently, a pair of ZFNs needs to be engineered for each target site and these must be oriented correctly to allow dimerization. Zinc-finger-DNA binding domains can be engineered for novel target sites using a variety of strategies, including phage display, modular assembly, bacteria-based two-hybrid and one-hybrid systems, and combination approaches [32]. Engineering ZFNs that have high activity and high specificity to endogenous target sites remains challenging, although ZFN design strategies are continually improving. The best-quality ZFNs have been made by Sangamo using a combination of phage display and modular display first developed by Klug and his co-workers [111] followed by rational design. These ZFNs have entered clinical trials in which the engineered T cells have been shown to be safe [65].

**TAL effector nucleases (TALENs)** are also artificial proteins. They share a similar structure to ZFNs in which an engineered DNA-binding domain is fused to the nuclease domain from FokI [36]. In TALENs, the DNA-binding domain is engineered by assembling a series of TAL repeats, with each repeat mediating interaction with a single base through a two-amino acid repeat variable di-residue (RVD) that can be described by a simple code [112, 113]. Thus, creating a highly active TALEN is much simpler than creating a highly active ZFN and simply involves using the code to assemble the correct TAL repeats needed to recognize a novel target sequence. In addition to the TAL repeats using natural RVDs, TAL repeats using engineered RVDs are now being used to create a TALEN [91]. These engineered RVDs might have increased specificity over natural RVDs, although that remains to be further studied. As for ZFNs, a pair of TALENs needs to be engineered to recognize a single target site. Even TALENs that use TAL repeats containing natural RVDs may have better specificity than ZFNs.

**CRISPR/Cas9 nucleases** (unfortunately, there is no agreement on a shorter abbreviation as CRISPR is already an abbreviation of ‘Clustered Regularly Interspaced Short Palindromic Repeats’) are derived from a bacteria-based adaptive immune system [114, 115]. In contrast to the other three platforms, the CRISPR/Cas9 nuclease system does not derive specificity through protein–DNA interaction but instead through RNA–DNA Watson-Crick base pairing. In the CRISPR/Cas9 system, a single-guide RNA (sgRNA) is designed such that the 20-bp recognition region of the sgRNA is identical to the desired target site (for Cas9, this 20-bp sequence is derived from *Streptococcus pyogenes*). The target site must be adjacent to a proto-spacer adjacent motif (PAM) sequence, which the Cas9 protein uses to identify target sites [115]. The multifunctional Cas9 protein, in complex with the sgRNA, is able to unwind double-stranded DNA, to interrogate whether the guide strand is sufficiently identical to the target site (small mismatches and bulges are tolerated [92, 116–119]) and then to create a blunt DSB if there is sufficient identity. Thus, CRISPR/Cas9 nucleases can be engineered very easily and between a third and a half of designed nucleases seem to be active at their desired target site.

In addition to the four basic platforms described above, other nucleases have been engineered to recognize therapeutically relevant human target sites. In **Mega-TALs**, a re-engineered meganuclease is fused to a small number of TAL effector repeats in order to increase binding affinity [41, 49]. In **Cas9-Fn** fusions, a nuclease inactive Cas9 protein is fused to the FokI nuclease (Fn) domain [42, 43, 93]. Like ZFNs and TALENs, the Cas9-Fn platform requires a pair of nucleases to be engineered to cut a specific target site. Finally, the proof-of-concept work of Roth and co-workers [120] showed that nickases could stimulate gene targeting, and so nickase versions of the nuclease platforms have been studied. The nickase versions may have improved specificity because they are associated with a decreased probability of generating an insertion/deletion at an off-target site, but they are usually 10-fold or more less active in stimulating HR-mediated genome editing at the on-target site. Thus, nickase versions may not have sufficient on-target editing activity to be therapeutically viable. The size of the relevant transcripts is an important consideration in determining the ease with which the platform might be packaged into various delivery platforms. For example, recombinant adeno-associated virus (AAV) has a packaging limit of 4.7 kilobases which is too small to package a pair of TALENs or the Cas9 cDNA of *S. pyogenes*, but not of *Staphylococcus aureus* [70].

## Abbreviations

AAV: Adeno-associated virus
allo-HSCT: Allogeneic hematopoietic stem cell transplantation
bp: Base pairs
CRISPR: Clustered regularly interspaced short palindromic repeats
DSB: Double-stranded break
FAH: Fumarylacetoacetate hydrolase
Fn: FokI nuclease
HR: Homologous recombination
HSPC: Hematopoietic stem/progenitor cell
NHEJ: Nonhomologous end-joining
RNAi: RNA interference
RNP: Ribonucleoprotein
RVD: Repeat variable di-residue
sgRNA: Single-guide RNA
ssODN: Single-stranded oligonucleotide
TALEN: TAL effector nuclease
ZFN: Zinc-finger nuclease
